# Transcription Factor *CaSBP12* Negatively Regulates Salt Stress Tolerance in Pepper (*Capsicum annuum* L.)

**DOI:** 10.3390/ijms21020444

**Published:** 2020-01-10

**Authors:** Huai-Xia Zhang, Wen-Chao Zhu, Xiao-Hui Feng, Jing-Hao Jin, Ai-Min Wei, Zhen-Hui Gong

**Affiliations:** 1 College of Horticulture, Northwest A&F University, Yangling 712100, China; huaixia@nwsuaf.edu.cn (H.-X.Z.); fengjn1555@163.com (X.-H.F.); Jinjinghao123@126.com (J.-H.J.); 2 Guizhou Institute of Pepper, Guizhou Academy of Agricultural Sciences, Guiyang 550009, China; chaochao5115.com@163.com; 3 Tianjin Vegetable Research Center, Tianjin Academy of Agricultural Sciences, Tianjin 300192, China; waimin163@163.com

**Keywords:** pepper, *CaSBP12*, salt stress, reactive oxygen species, *Nicotiana benthamiana*

## Abstract

SBP-box (Squamosa-promoter binding protein) genes are a type of plant-specific transcription factor and play important roles in plant growth, signal transduction, and stress response. However, little is known about the role of pepper SBP-box transcription factor genes in response to abiotic stress. Here, one of the pepper SBP-box gene, *CaSBP12*, was selected and isolated from pepper genome database in our previous study. The *CaSBP12* gene was induced under salt stress. Silencing the *CaSBP12* gene enhanced pepper plant tolerance to salt stress. The accumulation of reactive oxygen species (ROS) of the detached leaves of *CaSBP12*-silenced plants was significantly lower than that of control plants. Besides, the Na^+^, malondialdehyde content, and conductivity were significantly increased in control plants than that in the *CaSBP12*-silenced plants. In addition, the *CaSBP12* over-expressed *Nicotiana benthamiana* plants were more susceptible to salt stress with higher damage severity index percentage and accumulation of ROS as compared to the wild-type. These results indicated that *CaSBP12* negatively regulates salt stress tolerance in pepper may relate to ROS signaling cascades.

## 1. Introduction

Plants frequently encounter stressful environmental conditions, such as high salinity, extreme temperatures, drought, pests, and fungus infection [1]. These leading to detrimental effects on plant growth and seed germination and fruit productivity [2]. Salinity is the principal cause of crop yield loss worldwide and adversely affects plant growth and productivity [3]. To prevent the potentially harmful effects of such stresses, plants have evolved complex mechanisms to recognize external signaling networks and to be evidence of adaptive responses at the physiological, biochemical, and molecular levels [3,4]. In these responses, a series of stress-responsive genes are induced in plants, largely regulated by a range of transcription factors [5]. To date, a lot of transcription factors from various plant species have been reported to be involved in stress responses [6]. For instance, the basic leucine zipper transcription factor *AtbZIP24* [7], the *WRKY18*, *WRKY25*, *WRKY33*, *WRKY60*, and *WRKY63* [8,9,10], the *MYC2* and *MYB15* [11,12] have been reported to be involved in abiotic stress in *Arabidopsis*. The *NbWRKY79* has been reported to be involved in salt stress in *Nicotiana benthamiana* (*N. benthamiana*) [3]. The *SlGRAS40* and *SlAREB* have been reported to be involved in abiotic stress in tomato [2,13].

SBP-box (Squamosa-promoter binding protein) genes are specific to plants, which contain a highly conserved SBP domain [14]. This SBP domain comprises approximately 76 amino acid residues, including two zinc fingers (C3H and C2HC) and a nuclear localization signal [15,16]. Many studies show that SBP-box genes are involved in the network of the flower formation pathway, plant growth and development. For example, *AmSBP1* and *AmSBP2* can interact with the promoter sequence of the floral meristem identity gene SQUAMOSA and control of early flower development in *Antirrhinum majus* [14]. The SOC1-SPL module integrates photoperiod and gibberellic acid signals to control flowering time in *Arabidopsis* [17]. *OsSPL9* can directly bind to the CuRE element in the promoter region of *miR528* gene and activate the transcription of *miR528*, which regulates rice development at heading stage [18]. However, some studies show that SBP-box genes play a role in responses to abiotic and biotic stresses. For example, *AtSPL14* is induced by the fungal toxin fumonisin B1, which induces programmed cell death in *Arabidopsis* [19]. *CaSBP12* involved in plant defense response to *Phytophthora capsici* infection in pepper plants [20]. *OsSPL9* can activate the transcription of *miR528* gene, promote its accumulation in rice, then inhibit the expression of target mRNA AO, and finally eliminate the inhibition of ascorbic acid oxidase (AO) on RSV [21]. Knockout of *OsSPL10* in rice enhanced plants’ salt tolerance and overexpression of *OsSPL10* in rice reduced plants’ salt tolerance [22]. Overexpression of *BpSPL9* in *Betula platyphylla* Suk. improved the ability to scavenging of ROS to salt stress [23]. *slSBP2-L1* (Solyc05g015840) was highly induced by salt stress in tomato [24]. *Arabidopsis* miR156 regulates tolerance to heat stress through the transcription factors of *SPL2*, *SPL9*, and *SPL11* [25]. Overexpression of *OsmiR156k* in rice reduced the tolerance to cold stress by down-regulating *SPL3*, *SPL14*, and *SPL17* [26]. Knocking down a *microRNA156* target gene, *SPL13*, improves drought tolerance in alfalfa (*Medicago sativa*) [27].

However, to our knowledge, there is no report on the research of *CaSBP* genes in abiotic stress in pepper. In our previous study, we identified a *CaSBP12* gene (Accession no. Capana10g000886), which encodes 299 amino acids, contains a SBP domain, and is located in the nucleus [16,20]. Besides, our previous work indicates that *CaSBP12* gene negatively regulates plants defense response to *Phytophthora capsici* infection in pepper [20]. However, the role of *CaSBP12* in abiotic stress is not clear. In this study, we found that *CaSBP12* gene responses to salt stress, and plays a negative role in the tolerance to salt stress in pepper.

## 2. Results

### 2.1. Expression of the CaSBP12 Gene in Pepper under Salt Stress

To identify whether *CaSBP12* gene is involved in the response to salt stress, the expression pattern of *CaSBP12* in pepper were investigated. As shown in the Supplementary Figure S1, the expression of *CaSBP12* was induced at 2 h after salt stress. However, the expression level of *CaSBP12* was significantly decreased at 8 h and 12 h. These results indicated that *CaSBP12* gene is related to salt stress.

### 2.2. Silencing the CaSBP12 Gene Enhanced Pepper Plant Tolerance to Salt Stress

**Figure 1:**
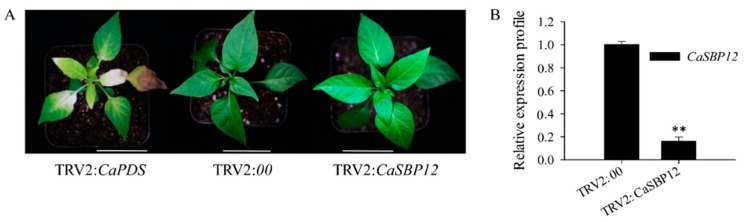
Phenotypes and silencing efficiency of *CaSBP12* in silenced and control plants. (**A**) The phenotypes of CaSBP12-silenced plants. Photographs were taken 40 days post-infiltration. The white line is used as a scale bar (length 3.5 cm) (the diameter of the pot is 7 cm). (**B**) Silencing efficiency of *CaSBP12* in CaSBP12-silenced plants. ** Represents significant differences at *p* ≤ 0.01. Mean values and SDs for three biological replicates are shown.

To identify whether the *CaSBP12* gene involved in salt stress, *CaSBP12* gene was silenced in pepper using the virus-induced gene silencing method [28]. In this study, a positive control vector (TRV2:*CaPDS*) was used for the silencing of the *CaPDS* gene (GenBank accession number: X68058), which exhibited photo-bleaching phenotype in the leaves after it was silenced, while the negative control was TRV2:*00*. When the leaves of TRV2:*CaPDS* plants exhibited the photo-bleaching phenotype (Figure 1A), the silencing efficiency of TRV2:*CaSBP12* and TRV2:*00* were detected. As shown in Figure 1A and Supplementary Figure S2, there were no visual phenotype and total chlorophyll content differences between CaSBP12-silenced (TRV2:*CaSBP12*) and control (TRV2:*00*) plants under normal conditions, and the silencing efficiency was over 80% (Figure 1B). Subsequently, CaSBP12-silenced and control plants were used for further study.

**Figure 2:**
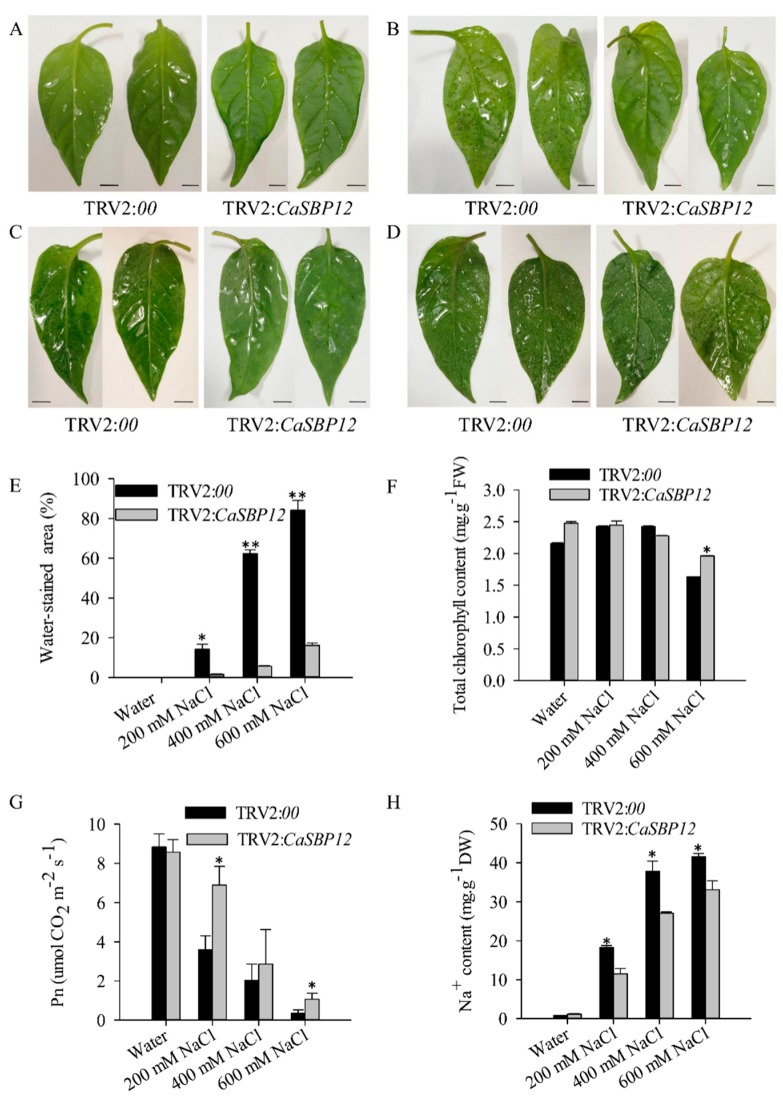
Phenotypes of the detached leaves of CaSBP12-silenced and control plants after 2 days of salt stress. (**A**) Phenotypes of the detached leaves of silenced plants after 2 days of treatment with water. (**B**) Phenotypes of the detached leaves of silenced plants after 2 days of treatment with 200 mM NaCl. (**C**) Phenotypes of the detached leaves of silenced plants after 2 days of treatment with 400 mM NaCl. (**D**) Phenotypes of the detached leaves of silenced plants after 2 days of treatment with 600 mM NaCl. (**E**) Water-stained area (%) of the detached leaves of silenced plants after 2 days of treatment with salt stress. (**F**) The total chlorophyll content of the detached leaves of silenced plants after 2 days of treatment with salt stress. (**G**) The net photosynthetic rate (Pn) of the detached leaves of silenced plants after 2 days of treatment with salt stress. (**H**) The Na^+^ content of the detached leaves of silenced plants after 2 days of treatment with salt stress. The black line used as a scale bar (length 0.5 cm). * and ** represent significant differences at *p* ≤ 0.05 and *p* ≤ 0.01 respectively. Mean values and SDs at least for three biological replicates are shown.

The detached leaves of CaSBP12-silenced and control plants were soaked in different concentrations of salt solution (200 mM NaCl, 400 mM NaCl, and 600 mM NaCl). As shown in Figure 2A, there was no phenotypic difference between the detached leaves of CaSBP12-silenced and control plants treated in water for 2 days. However, there was an obvious phenotypic difference between the detached leaves of CaSBP12-silenced and control plants treated in 200 mM NaCl, 400 mM NaCl, and 600 mM NaCl solution for 2 days (Figure 2B–D). With the increase of salt treatment concentration, water-stained spots on the detached leaves increased and the area became larger. The detached leaves of control plants appeared water-stained spots, which accounted for about 14.19% of the leaf area after 2 days of treatment with 200 mM NaCl, while the water-stained spots on the detached leaves of CaSBP12-silenced plants only accounted for about 1.44% of the leaf area (Figure 2B,E). Besides, about 62.23% of the leaf area of the detached leaves of control plant was water-stained, while only about a 5.66% of the leaf area of the detached leaves of CaSBP12-silenced plant showed water-stained spots after 2 days of treatment with 400 mM NaCl (Figure 2C,E). In addition, about 84.15% of the leaf area of detached leaves of control plants were water-stained, while about 16.15% of leaf area of the detached leaves of CaSBP12-silenced plants were water-stained after 2 days of treatment with 600 mM NaCl (Figure 2D,E). After treated with 200mM NaCl and 400 mM NaCl for two days, there was no significant change in total chlorophyll content in the detached leaves of the CaSBP12-silenced plants compared with the control (Figure 2F). However, after 2 days of treatment with 600 mM NaCl, the total chlorophyll content of the detached leaves of the CaSBP12-silenced plant and the control plant was decreased, and the total chlorophyll content of the detached leaves of the CaSBP12-silenced plant was significantly higher than that of the control plant (Figure 2F). Besides, with the increase of salt treatment concentration, the net photosynthetic rate of the detached leaves of CaSBP12-silenced and control plants decreased (Figure 2G). However, the net photosynthetic rate of the detached leaves of CaSBP12-silenced plants was significantly higher than that of the control plants (Figure 2G). In addition, with the increase of salt treatment concentration, the Na^+^ content in the detached leaves of CaSBP12-silenced and control plants increased (Figure 2H). However, the Na^+^ content in the detached leaves of CaSBP12-silenced plants was significantly lower than that of control plants (Figure 2H). In addition, the expression of ion transport genes (*CaSOS1*, *CaHKT2-1*, *CaHKT2-2*) were suppressed in the CaSBP12-silenced plants without any treatment (Supplementary Figure S3). These results indicated that silencing the *CaSBP12* gene enhanced pepper plant tolerance to salt stress.

**Figure 3:**
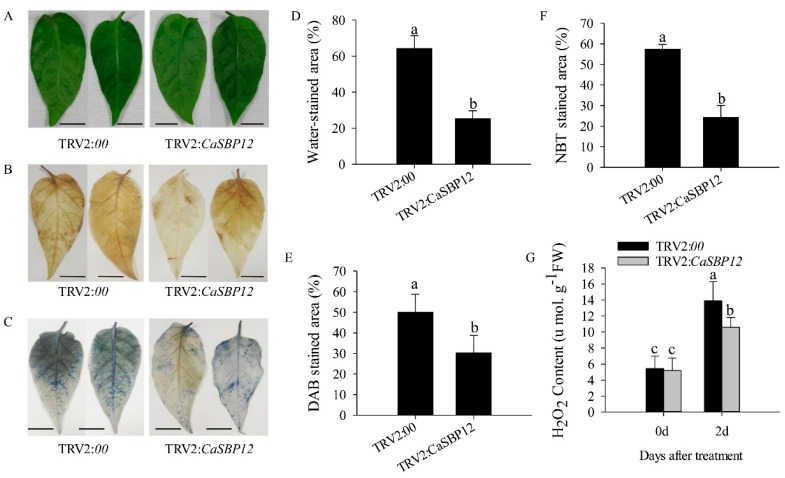
DAB and NBT staining of silenced plants and the phenotypes of the detached leaves of silenced plants. (**A**) Phenotypes of the detached leaves of CaSBP12-silenced and control plants after 2 days of treatment with 400 mM NaCl. (**B**) DAB staining in leaves of CaSBP12-silenced and control plants after 2 days of treatment with 400 mM NaCl. (**C**) NBT staining in leaves of CaSBP12-silenced and control plants after 2 days of treatment with 400 mM NaCl. (**D**) Water-stained area (%) of the detached leaves of silenced plants after 2 days of treatment with 400 mM NaCl. (**E**) The DAB stained area (%) of the detached leaves of silenced plants after 2 days of treatment with 400 mM NaCl. (**F**) The NBT stained area (%) of the detached leaves of silenced plants after 2 days of treatment with 400 mM NaCl. (**G**) The H_2_O_2_ content of the detached leaves of silenced plants after 2 days of treatment with 400 mM NaCl. d: days. The black line is used as a scale bar (length 0.75 cm). Bars with different letters indicate significant differences at *p* ≤ 0.05. Mean values and SDs at least for three biological replicates are shown.

Additionally, in order to analyze the accumulation of ROS in the CaSBP12-silenced and control plants after salt stress, DAB and NBT staining were used to detect peppers hydrogen (H_2_O_2_) and O_2_^−^ levels (Figure 3). As shown in Figure 3A–E, after 2 days of salt stress, about 64.28% of leaf area of water-stained lesions appeared in the detached leaves of the control plants, while about 25.24% of leaf area of detached leaves of the CaSBP12-silenced plants was water-stained. The DAB stained area was significantly increased in the detached leaves of control plants than that in the detached leaves of CaSBP12-silenced plants. Besides, as shown in Figure 3C,F, the NBT stained area was significantly increased in the detached leaves of control plants than that in the detached leaves of CaSBP12-silenced plants. In addition, the H_2_O_2_ content in the detached leaves of control plants was significantly higher than that in the detached leaves of CaSBP12-silenced plants (Figure 3G). These results indicated that lower accumulation of H_2_O_2_ and O_2_^−^ was detected in the detached leaves of CaSBP12-silenced plants compared with control plants.

**Figure 4:**
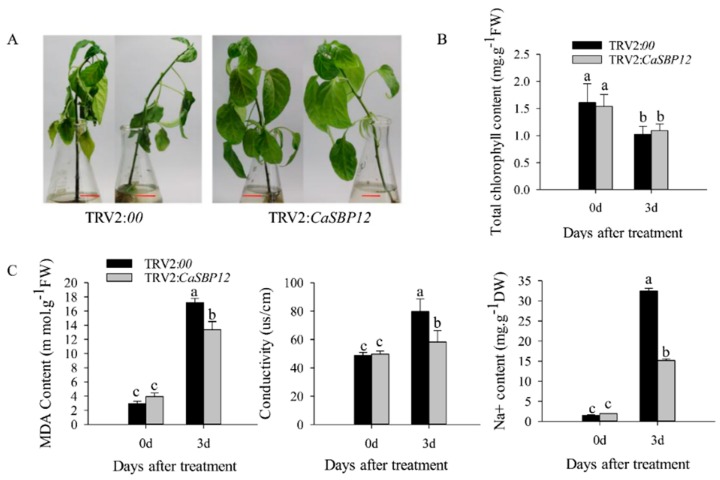
Phenotypes, total chlorophyll, Na^+^, malondialdehyde (MDA) content and conductivity of silenced plants after 3 days of salt stress. (**A**) Phenotypes of CaSBP12-silenced and control plants after 3 days of treatment with 400 mM NaCl. The red line is used as a scale bar (length 2 cm). (**B**) The total chlorophyll content of CaSBP12-silenced and control plants after 3 days of treatment with 400 mM NaCl. (**C**) The Na^+^, MDA content, and conductivity of CaSBP12-silenced and control plants after 3 days of treatment with 400 mM NaCl. d: days. Bars with different letters indicate significant differences at *p* ≤ 0.05. Mean values and SDs for three biological replicates are shown.

In order to further verify the role of *CaSBP12* gene under salt stress, the CaSBP12-silenced and control plants were treated with 400 mM NaCl. After 3 days of treatment, wilted and yellowish symptoms were observed in the control plants, while the CaSBP12-silenced plants were only yellowish (Figure 4A). The total chlorophyll content of the CaSBP12-silenced and control plants were decreased (Figure 4B). However, there was no significant difference in total chlorophyll content between control and CaSBP12-silenced plants after 3 days of treatment (Figure 4B). Besides, the Na^+^, MDA content and conductivity were significantly increased in control plants than that in the CaSBP12-silenced plants (Figure 4C). These results indicated that silencing the *CaSBP12* gene enhanced pepper plant’s tolerance to salt stress.

**Figure 5:**
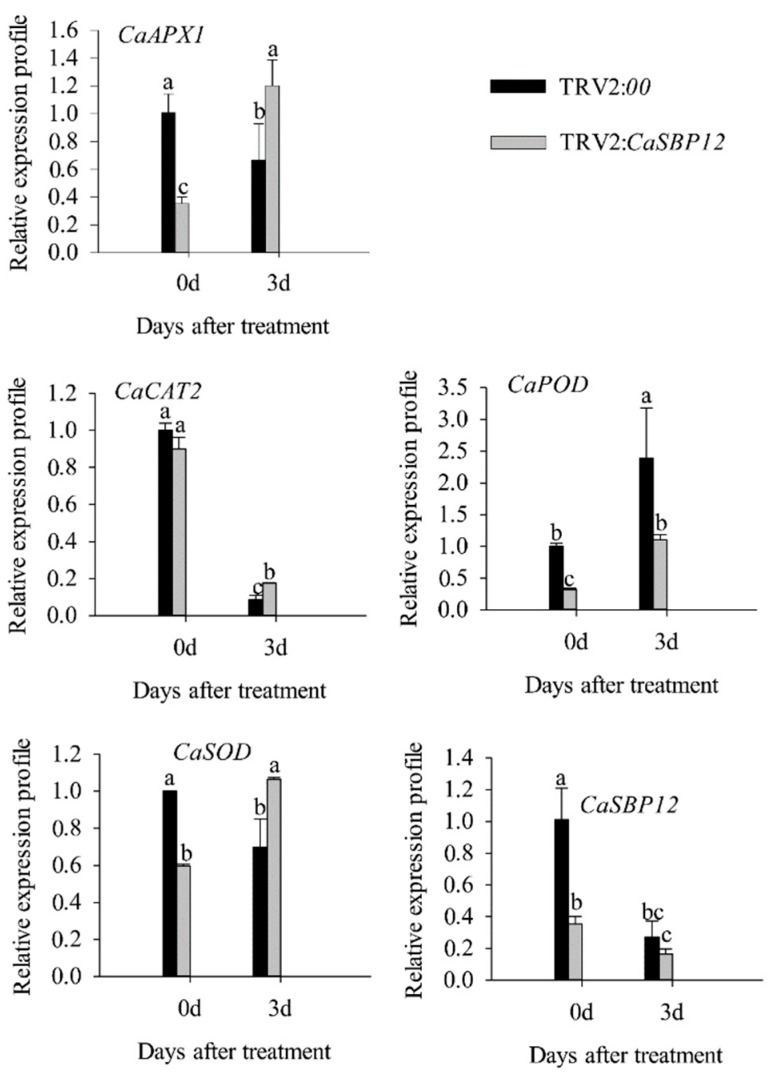
The expression of reactive oxygen species (ROS)-scavenging enzymes related genes after 3 days of salt stress (400 mM NaCl) in silenced plants. Bars with different letters indicate significant differences at *p* ≤ 0.05. Mean values and SDs for three biological replicates are shown.

In addition, the expression of *CaAPX1* (GenBank accession number: DQ002888.1), *CaCAT2* (GenBank accession number: AY128694.1), *CaSOD* (GenBank accession number: NM_001324998.1), and *CaPOD* (GenBank accession number: NM_001324997.1), which related to ROS-scavenging enzymes were detected. After 3 days of salt stress, the expression of these genes was induced in *CaSBP12*-silenced plants except for *CaCAT2* (Figure 5). The expression of *CaCAT2* was decreased in CaSBP12-silenced and control plants on day 3 (Figure 5). Besides, these genes, expression in CaSBP12-silenced plants was higher than that in control plants except the expression of *CaPOD* (Figure 5). In addition, the expression of *CaSBP12* was decreased in CaSBP12-silenced and control plants (Figure 5).

### 2.3. Overexpression of CaSBP12 in Nicotiana Benthamiana Enhanced Susceptibility to Salt Stress

**Figure 6:**
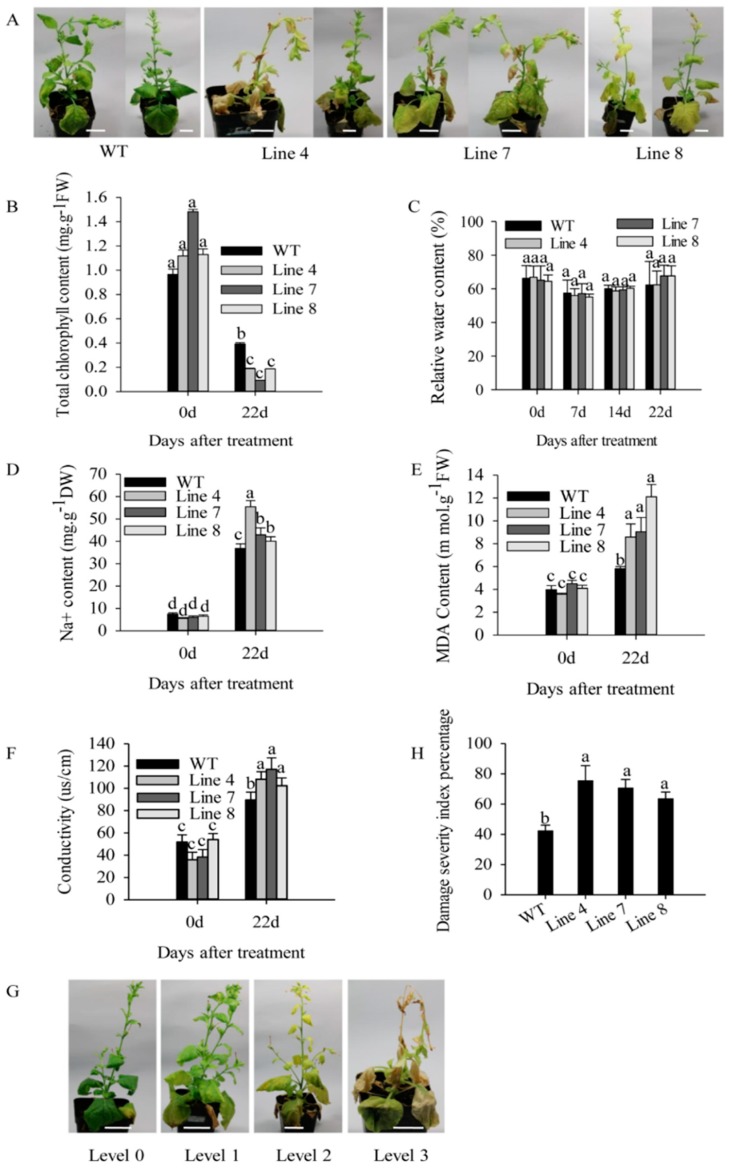
Overexpression of *CaSBP12* in *Nicotiana Benthamiana* enhanced susceptibility to salt stress. (**A**) Phenotypes of transgenic (Line 4, Line 7 and Line 8) and wild-type lines after 22 days of treatment with 200 mM NaCl. (**B**) The total chlorophyll content of transgenic and wild-type lines after 22 days of treatment with 200 mM NaCl. (**C**) The relative water content of transgenic and wild-type lines during treatment with 200 mM NaCl at day 0, day 7, day 14, and day 22. (**D**) The Na^+^ content of transgenic and wild-type lines after 22 days of treatment with 200 mM NaCl. (**E**) The MDA content of transgenic and wild-type lines after 22 days of treatment with 200 mM NaCl. (**F**) The conductivity of transgenic and wild-type lines after 22 days of treatment with 200 mM NaCl. (**G**) Classification of damage severity index percentage of transgenic and wild-type lines after 22 days of treatment with 200 mM NaCl. Level 0: no symptoms; Level 1: yellowing of lower leaves of plants; Level 2: the whole plant is yellowing and the edge of leaves is decolorized seriously; Level 3: whole plant yellowing and growth point death. (**H**) Damage severity index percentage of transgenic and wild-type lines after 22 days of treatment with 200 mM NaCl. The white line used as a scale bar (length 3.5 cm). Bars with different letters indicate significant differences at *p* ≤ 0.05. Mean values and SDs for three replicates are shown.

In order to further confirm the role of *CaSBP12* in plant defense response to salt stress, we generated transformed *Nicotiana benthamiana* plants overexpressing *CaSBP12*, as the stable transformation of pepper plants remains challenging. Three transgenic lines were randomly selected for this study. The *CaSBP12* gene was significantly expressed in transgenic lines as compared with wild-type (WT) (Supplementary Figure S4). After 22 days of salt treatment, yellowing appeared in the leaves of both transgenic and wild-type lines (Figure 6A). However, almost the whole leaves of transgenic lines was severe yellowed or some of them appeared albinism, while the yellowing of wild-type lines was not obvious, or only the lower leaves were yellowing (Figure 6A). Besides, the total chlorophyll content of transgenic lines was significantly lower than that of wild-type lines (Figure 6B). During salt stress, there was no difference in relative water content between transgenic and wild-type lines, as compared with those without salt stress (Figure 6C). The Na^+^, MDA content, and conductivity were significantly increased in transgenic and wild-type lines, and they were higher in the transgenic lines than that in the wild-type lines (Figure 6D–F). However, there was no significant difference in the expression of *NbSOS1* (ion transport gene) between transgenic and wild-type lines without any treatment (Supplementary Figure S5). After 22 days of salt stress, the damage symptoms in the transgenic (line 4, line 7, and line 8) and wild-type plants were divided into four levels. Level 0: no symptoms; Level 1: yellowing of lower leaves of plants; Level 2: the whole plant is yellowing and the edge of leaves is decolorized seriously; Level 3: whole plant yellowing and growth point death (Figure 6G). Then, the damage severity index percentage was calculated. As shown in Figure 6H, the damage severity index percentage of transgenic lines was significantly higher than that of wild-type lines. The detail data of damage severity index percentage was supplied in Supplementary Table S1.

**Figure 7:**
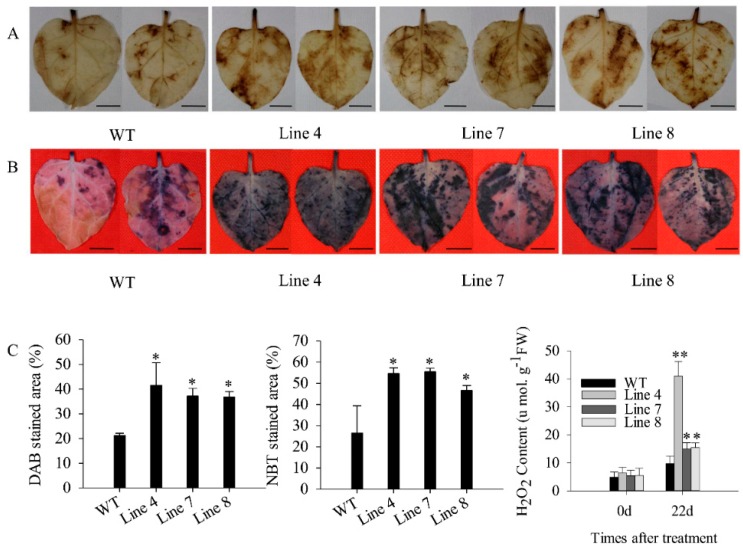
DAB and NBT staining of transgenic and wild-type lines after 22 days of salt stress. (**A**) DAB staining of transgenic and wild-type lines after 22 days of treatment with 200 mM NaCl. (**B**) NBT staining of transgenic and wild-type lines after 22 days of treatment with 200 mM NaCl. (**C**) The DAB, NBT stained area (%) and H_2_O_2_ content of transgenic and wild-type lines after 22 days of treatment with 200 mM NaCl. The black line is used as a scale bar (length 0.75 cm). * and ** represent significant differences at *p* ≤ 0.05 and *p* ≤ 0.01 respectively. Mean values and SDs at least for three biological replicates are shown.

**Figure 8:**
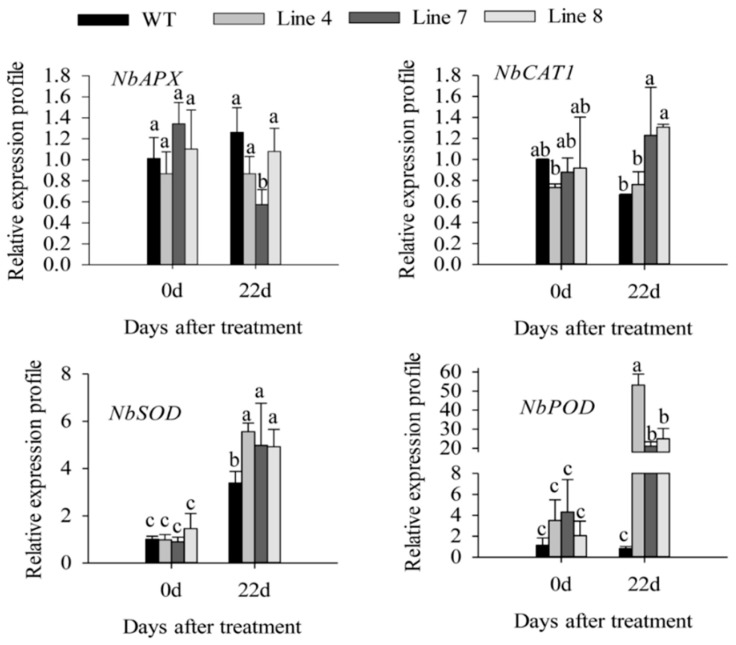
The expression of ROS-scavenging enzymes related genes in transgenic and wild-type lines after 22 days of salt stress. Bars with different letters indicate significant differences at *p* ≤ 0.05. Mean values and SDs for three biological replicates are shown.

Besides, the DAB and NBT stained area of transgenic lines was significantly larger than that of wild-type lines (Figure 7A–C). In addition, the H_2_O_2_ content in the transgenic lines was significantly higher than that of wild-type lines (Figure 7C). After 22 days of salt stress, the expression of *NbSOD* (SGN locus: Niben101Scf09401g00007.1) and *NbPOD* (SGN locus: Niben101Scf01124g05002.1) was induced in the transgenic lines, and was higher than that in wild-type lines (Figure 8). However, the expression of *NbAPX* (GenBank accession number: AB610799.1) and *NbCAT*1 (GenBank accession number: EU998969.1) showed no significant change compared with day 0 (Figure 8). These results indicated that overexpression of *CaSBP12* in *Nicotiana Benthamiana* enhanced susceptibility to salt stress.

## 3. Discussion

The SBP-box gene family is composed of plant-specific transcription factors encoding proteins that contain a highly conserved SBP domain [14]. They play significant roles in plant growth and development, biosynthesis of gibberellic acid (GA), endoplasmic reticulum (ER) stress signaling, and response to biotic and abiotic stress [19,23,29,30,31]. Although these functions of the SBP-box gene have been investigated to some extent in other plants, the function of SBP-box genes in pepper, especially in abiotic stress has not been studied so far.

Previous studies have shown that *CaSBP12* gene is located in the nucleus and plays a negative role in the defense response of pepper to *Phytophthora capsici* infection [20]. Here we found that it can be induced at the early stage of salt stress and inhibited in the later stage (Supplementary Figure S1). In order to identify whether the *CaSBP12* gene is involved in salt stress, we silenced it in pepper plants. There is no phenotypic difference between CaSBP12-silenced and control plants under normal conditions (Figure 1A). It has been reported that most of the SBP-box genes play an important role in plant morphogenesis, growth, and development. For example, knockout *AtSPL8* affects megasporogenesis, trichome formation on sepals, and stamen filament elongation in *Arabidopsis* [32]. The petiole and leaf margin serration of *Arabidopsis* was elongated and enhanced respectively, as the mutation of *AtSPL14* [19]. However, there was no phenotypic difference between CaSBP12-silenced and control plants. This may be due to the fact that gene silencing does not completely disable the function of the gene as gene mutation does, or that *CaSBP12* does not play a role in pepper growth and development. However, this requires further experimental verification. The detached leaves of CaSBP12-silenced plants were more tolerant to salt stress than those of control plants (Figure 2). Besides, with the increase of salt concentration, the accumulation of Na^+^ also increased, while the net photosynthetic rate was decreased (Figure 2). Salt stress has three-fold effects, viz., it reduces water potential and causes ion imbalance or disturbances in ion homeostasis and toxicity [33]. Salt toxicity is one of the reasons for the decrease of photosynthetic rate [33]. In addition, we found that the expression of ion transport genes (*CaSOS1*, *CaHKT2-1*, and *CaHKT2-2*) in CaSBP12-silenced plants was suppressed without any treatment (Supplementary Figure S3). However, there was no significant difference in the expression of *NbSOS1* between transgenic and wild-type lines without any treatment (Supplementary Figure S5). Therefore, *CaSBP12* may suppress Na+ uptake in *CaSBP12*-silenced plants by inhibiting the expression of sodium transport gene, but this needs further experimental verification. The accumulation of H_2_O_2_ and O_2_^−^ in the detached leaves of CaSBP12-silenced plants was less than that in the detached leaves of control plants (Figure 3). It has been reported that abiotic stress is related to ROS signaling pathway. For example, overexpression of *VpSBP16* in *Arabidopsis* enhances tolerance of salt stress during seed germination, as well in seedlings and mature plants, by regulating ROS signaling cascades [4]. Overexpression of *BpSPL9* improved scavenging of ROS by activation of peroxidase (POD) and superoxide dismutase (SOD) enzymes, and enhanced the plant’s tolerance to salt and drought stress [23]. *CaHsp25.9* may play a positive role in reducing the accumulation of ROS, and positively regulate the tolerance to heat, salt, and drought stress in pepper plants [1]. Besides, it has been reported that superoxide dismutase (SOD), catalase (CAT), ascorbate peroxidase (APX), glutathione peroxidase (GPX), and perox-iredoxin (PrxR) is the major ROS-scavenging enzymes of plants [34]. In addition, the “classical” plant peroxidases (class III) regulates the scavenging of H_2_O_2_ [34,35,36]. Therefore, we detected the expression of *CaAPX1*, *CaCAT2*, *CaSOD*, and *CaPOD*, which are related to ROS-scavenging enzymes after 3 days of salt stress. The expression of these genes was induced in *CaSBP12*-silenced plants except for *CaCAT2* (Figure 5). The expression of *CaCAT2* was decreased in CaSBP12-silenced and control plants on day 3 (Figure 5). Moreover, these genes, expression in CaSBP12-silenced plants was higher than that in control plants, except for the expression of *CaPOD* (Figure 5). In addition, the expression of *CaSBP12* was decreased in CaSBP12-silenced and control plants (Figure 5). It has been reported that there is a strong link between ROS signaling, the redox network of cells, and the different antioxidant pools in different cells [37]. The double mutant deficient in APX1 and CAT2 in *Arabidopsis*, and APX1 and CAT1 in tobacco (*Nicotiana tabacum*) is more tolerant to different environmental conditions compared with wild type, and the single apx or cat mutants [38,39]. In order to further confirm the role of *CaSBP12* in plant defense response to salt stress, we generated transformed *N. benthamiana* plants overexpressing *CaSBP12.* The overexpression of *CaSBP12* transgenic lines was more sensitive to salt stress compared to wild-type lines with a higher damage severity index percentage (Figure 6). Besides, after 22 days of salt stress, the transgenic lines showed serious yellowing, and some leaves even showed albinism and death (Figure 6A). In addition, the accumulation of Na^+^ in the transgenic lines was more than that in the wild-type lines (Figure 6D). It has been reported that the mechanisms of salt stress damage include osmotic stress and ion toxicity [40]. The high concentration of salt in the soil reduces the water potential of the soil, makes it difficult for plants to absorb water, and even causes the water in plants to seep out, thus causing water deficit and osmotic stress in plants [40]. However, in this study, with the increase of salt treatment time, the relative water content of transgenic lines did not change significantly (Figure 6C). Besides, severe salt stress usually inhibits plant growth and even causes plant death [33]. Therefore, after 22 days of salt stress, it is mainly ion toxicity. The accumulation of ROS in the transgenic lines was more than that in the wild-type lines (Figure 7). The expression of *NbPOD* and *NbSOD* was highly induced, especially in transgenic lines (Figure 8). It is worth noting that the expression of *CaSOD* in the CaSBP12-silenced plants is higher than that in the control plants after 3 days of salt treatment (Figure 5), which is not consistent with the trend of the expression of *NbSOD* in transgenic lines. This may be due to the complex defense system evolved by plants themselves against biotic and abiotic stresses. In addition, after abiotic stress, plants produce active oxygen that activates some active oxygen scavenging enzymes. The APX, CAT, POD, and SOD are major active oxygen scavenging enzymes. SOD converts superoxide radicals into hydrogen peroxide, CAT and POD decompose hydrogen peroxide into water, these three enzymes form a complete anti-oxidation chain [41]. It has been reported that overexpression of *BpSPL9* in *Betula platyphylla* Suk. improved the ROS scavenging ability under salt stress though the activation of POD and SOD [23]. Silencing the PEROXIDASE2, *CaPO2*, in pepper compromised H_2_O_2_ accumulation, both locally and systemically, during avirulent Xcv infection [42]. Besides, *CaPO2* peroxidase is involved in ROS generation, both locally and systemically, to activate cell death and PR gene induction during the defense response to pathogen invasion [42]. Therefore, we speculate that *CaSBP12* regulating plant tolerance to salt stress may be related to ROS, and this needs further experimental verification.

## 4. Materials and Methods

### 4.1. Plant Materials and Growth Conditions

Pepper cultivar AA3 provided by the Capsicum Research Group, College of Horticulture, Northwest A&F University, P. R. China and *N. benthamiana* were used in this study. Pepper plants were grown in a growth chamber at 22 °C (day for 16 h)/18 °C (night for 8 h) and a relative humidity of 80%. *N. benthamiana* plants were grown in a growth chamber at 25 °C (day for 16 h)/18 °C (night for 8 h) and a relative humidity of 60%.

### 4.2. Virus-Induced Gene Silencing (VIGS) of CaSBP12 Gene in Pepper

The tobacco rattle virus (TRV)-based VIGS system was used for silencing the *CaSBP12* gene in pepper, following the method described by Wang (2013) [28]. To generate the VIGS plasmid construction of *CaSBP12* gene, a 250-bp fragment of *CaSBP12* was amplified using their specific primer (Supplementary Table S2). The obtained product was cloned into the TRV2 vectors using the double digested method with *Bam*HI and *Kpn*I enzymes. Then, it was sequenced through Sangon Biotech Company (Shanghai, China). The recombined vector (TRV2:CaSBP12), TRV2:00 (negative control), TRV2:CaPDS (phytoenedesaturase, positive control), and TRV1 were transformed into *Agrobacterium tumefaciens* strain GV3101 using the freeze-thaw method. Pepper seedlings at two true leaves stage were used for the silencing of *CaSBP12* gene using the method described by Zhang et al. (2013) [43]. The injected plants were first grown in a growth chamber at 18 °C in dark for 2 days, and then grown in a growth chamber at 22 °C (day for 16 h)/18 °C (night for 8 h) with 60% relative humidity. Forty days post-infiltration, leaves from the control and silenced plants were collected to measure the silencing efficiency.

### 4.3. Nicotiana Benthamiana Transformation

The full encoding regions (900-bp) of *CaSBP12* was cloned into the PBI121:GUS vector with *Xba*I and *Bam*HI restriction enzyme sites to yield the final plasmid PBI121:CaSBP12:GUS for genetic transformation (Supplementary Table S2). Over expression lines of *CaSBP12* were obtained by *Agrobacterium*-mediated tobacco leaf disc transformation method [44]. Three kanamycin-resistant lines of transgenic *N. benthamiana* plants harboring the PBI121:CaSBP12:GUS construct were selected. Besides, they were confirmed using quantitative real-time PCR during T1 generation (Supplementary Table S3). Seeds of T1 plants were obtained from regenerated T0 plants, and seedlings of T2 lines were further selected on MS agar plates containing 50µg/mL kanamycin. T3 plants were used for further analyses.

### 4.4. Stress Treatments and Samples Collection

For detecting the expression of *CaSBP12* gene in pepper under salt stress, pepper plants at the stage of 6–8 true leaves were removed from the substrate (matrix, vermiculite, and perlite mixed at 3:1:1 ratio) and cultured in 1/2 Hoagland’s solution. After culturing for 5 days, the plants were treated with 400 mM NaCl with 1/2 Hoagland’s solution. The control plant was cultured in 1/2 Hoagland’s solution only [45]. Leaves were harvested at 0 h, 2 h, 4 h, 8 h, 12 h, 24 h and store at −80 °C.

For salt stress, the detached leaves of TRV2:CaSBP12 and control plants were treated with 200 mM NaCl, 400 mM NaCl, and 600 mM NaCl solution for 2 days. The detached leaves of TRV2:CaSBP12 and TRV2:00 plants were treated with 400 mM NaCl solution for 2 days. The plants of TRV2:CaSBP12 and control were soaked in 400 mM NaCl for three days [1,46]. Besides, the total chlorophyll content was measured, and the samples were collected at day 0 and day 3 and stored at −80 °C.

Salt stress on transgenic lines was done according to the modified method described by Liu et al. (2017) [2]. Each plant was planted in an independent basin (same basins were used), 11 or 10 plants of wild-type and *CaSBP12* transgenic lines were placed in a big pot and watered once a week to make sure the soil water in every basin was uniform and all the plants were grown in the same conditions. After three weeks, the wild-type and transgenic lines were watered with 200 mM NaCl every week (1000 mL per pot) for up to 22 days. The relative water content (RWC) and the total chlorophyll content was measured during treatment [47]. Samples were collected at day 0 and day 22, and stored at −80 °C.

### 4.5. RNA Extraction and Quantitative Real-Time PCR (qPCR)

Total RNA was isolated using the method described by Guo et al. (2012) [48]. The first chain was synthesized using Prime Script Kit (Takara, Dalian, China) following the manufacturer’s instructions. The cDNA concentration was diluted to 50 ng/µL and used for qPCR. Here, we provide the qPCR experiments as described by Bustin et al. (2009) [49]. The iQ5.0 Bio-Rad iCycler thermocycler (Bio-Rad, Hercules, CA, USA) machine was used for qPCR using the procedure described by Zhang et al. (2018) [20]. Briefly, the qPCR cycling conditions are as follows: pre-denaturation at 95 °C for 1 min, followed by 40 cycles of denaturation at 95 °C for 10 s, annealing at 56 °C for 30 s, and extension at 72 °C for 30 s. The fluorescent signal was measured at the end of each cycle, and melting curve analysis was performed by heating the PCR product from 56 to 95 °C in order to verify the specificities of the primers. The pepper actin mRNA, *CaActin2* (accession no. AY572427), was used as the reference in pepper [50]. The *Nicotiana benthamiana* actin gene, *Nbactin-97* (accession No. LOC109206422), was used as reference in *Nicotiana benthamiana* [51]. All the primer specificities used for the qPCR were evaluated using NCBI Primer BLAST (Supplementary Table S3). The details of the length of per gene amplification, the location of the primer were supplied in Supplementary Table S3. Gene expression was quantified following the 2^−△△CT^ method [52]. For example, the mean CT of the *CaSBP12* gene in untreated and treated samples was 26.34 and 29.64, respectively. The mean CT of the reference gene *(CaActin2*) in the untreated and treated samples was 20.78 and 21.41, respectively. The relative expression profile of *CaSBP12* can be calculated using the following formula:

The relative expression profile of *CaSBP12* = 2^(−[(29.64 − 21.41) − (26.34 − 20.78)])

### 4.6. Measurement of Physiological Indicators

The H_2_O_2_ and O_2_^−^ radical level was analyzed by DAB and NBT staining [53,54,55]. The quantification of the DAB, NBT stained area, and water-stained area was obtained using the method described by Sekulska-nalewajko et al. (2016) [56]. The H_2_O_2_ content was measured following the method described by Liu et al. (2010) [57]. The Na^+^ content was measured following the method described by Li (2015) [58]. The net photosynthetic rate (Pn) was measured using an American photosynthetic apparatus CIRAS-3. The malondialdehyde (MDA) and conductivity was measured using the method described by Ma et al. (2013) [20] and Kim et al. (2015) respectively [59]. The RWC were determined using the method described by Pan et al. (2012) [47]. The total chlorophyll content was measured using the method described by Arkus et al. (2005) [60].

### 4.7. Damage Severity Index Percentage Statistics

After 22 days of salt stress, the damage severity index percentage of plants was calculated. According to the damage severity of plants, the damage symptoms in the transgenic and wild-type plants were divided into four levels. They are as follows: Level 0: no symptoms; Level 1: yellowing of lower leaves of plants; Level 2: the whole plant is yellowing and the edge of leaves is decolorized seriously; Level 3: whole plant yellowing and growth point death. The damage severity index percentage was calculated using the following formula:

Damage severity index percentage = [(∑the numerical grade of damage × number of damage plants of this grade)/(the highest grade of damage × total number of surveys)] × 100

### 4.8. Statistical Analysis

Statistical analysis was performed using the data processing system (DPS7.05, China) for one-way analysis of variance (ANOVA) [20]. The value *p* ≤ 0.05 or *p* ≤ 0.01 was considered to be significantly different. All experiments were performed and analyzed separately with at least three biological replicates.

## 5. Conclusions

In a conclusion, the *CaSBP12* gene was induced by salt stress in pepper. Silencing the *CaSBP12* gene enhanced the tolerance to salt stress, and reduced the accumulation of ROS compared with the control plants in pepper. Overexpression *CaSBP12* gene in *N. benthamiana* enhanced plants’ sensitive to salt stress and the accumulation of ROS compared to wild-type plants. Besides, the expression of *NbPOD* and *NbSOD* was highly induced in transgenic plants than that in wild-type plants. These results indicated that *CaSBP12* plays a negatively role in plant tolerance to salt stress. Besides, *CaSBP12* regulating plant tolerance to salt stress may be related to ROS, and this needs further experimental verification.

## Supplementary Materials

Supplementary materials can be found at https://www.mdpi.com/1422-0067/21/2/444/s1.
