# Supplementary material for: Transcription Factor CaSBP12 Negatively Regulates Salt Stress Tolerance in Pepper (Capsicum annuum L.)
Source: Int J Mol Sci. 2020 Jan 10;21(2):444. doi: 10.3390/ijms21020444 (PMC7013666; doi:10.3390/ijms21020444)
Supplement: Supplementary file 1 [file ijms-21-00444-s001.pdf]

Figure S1. The expression profile of *CaSBP12* gene under salt stress in pepper. After 400 mM NaCl treatment, the expression of *CaSBP12* was detected at 0h, 2h, 4h, 8h, 12h, and 24h. h: hour. * and ** represent significant differences at P ≤ 0.05 and P ≤ 0.01 respectively. Mean values and SDs for three replicates are shown.

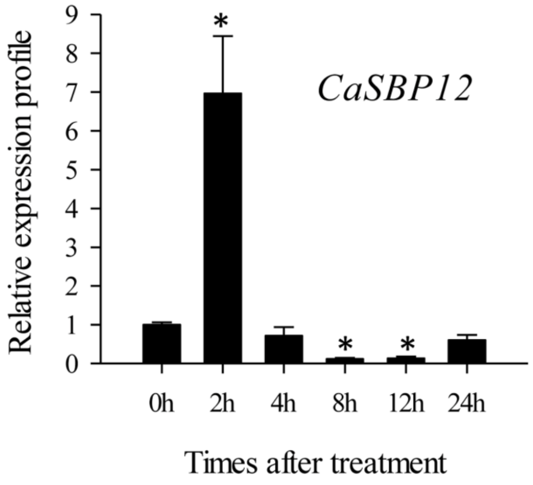

Figure S2. The total chlorophyll contents of CaPDS-silenced, CaSBP12-silenced, and control plants after forty days post-infiltration. Bars with different letters indicate significant differences at P ≤ 0.05. Mean values and SDs for three replicates are shown.

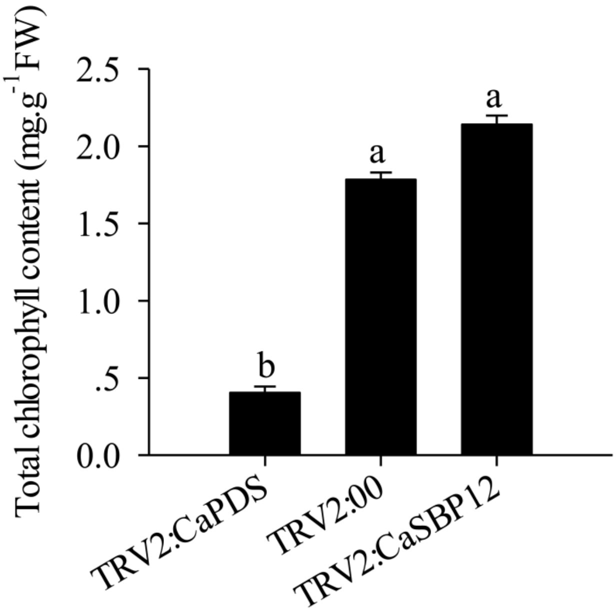

Figure S3. The expression level of ion transport genes in CaSBP12-silenced and control plants without any treatment were measured using quantitative real-time PCR. *CaSOS1*: CA08g01100; *CaHKT2-1*: CA07g09810; *CaHKT2-2*: CA01g10660. Bars with different letters indicate significant differences at P ≤ 0.05. Mean values and SDs for three replicates are shown.

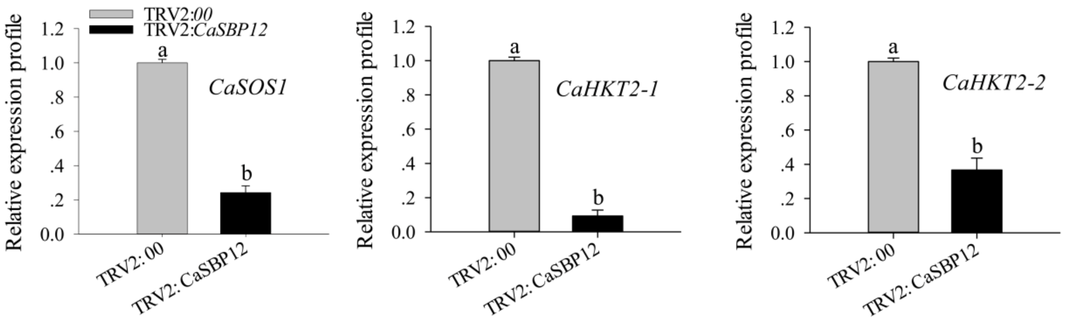

Figure S4. The expression of *CaSBP12* gene in transgenic lines and wild-type lines of *Nicotiana benthamiana*. The expression of *CaSBP12* gene in transgenic lines and wild-type lines of *Nicotiana benthamiana* was measured using quantitative real-time PCR. ** represent significant differences at P ≤ 0.01 respectively. Mean values and SDs for three replicates are shown.

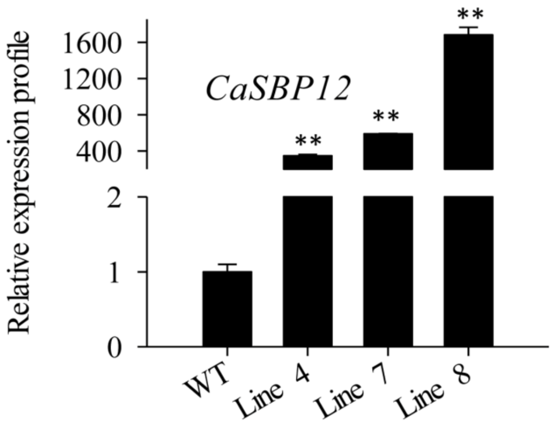

Figure S5. The expression level of *NbSOS1* (Niben101Scf02321g00027.1) gene in transgenic lines and wild-type lines of *Nicotiana benthamiana* was measured using quantitative real-time PCR without any treatment. Bars with different letters indicate significant differences at $P \leq 0.05$. Mean values and SDs for three biologic replicates are shown.

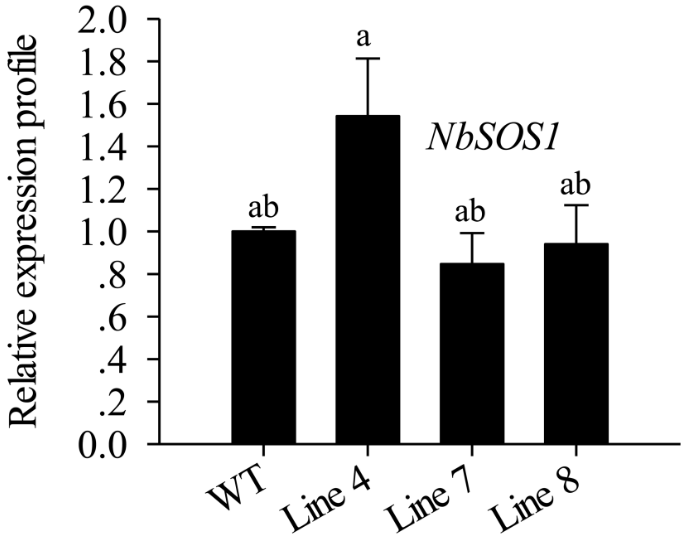

Table S1. The detail data of damage severity index percentage of transgenic (line 4, line 7, and line 8) and wild-type plants after salt stress twenty-two days.

	0 level plants	1 level plants	2 level plants	3 level plants	Total number of treatment plants	Damage severity index percentage
WT	3	8	8	1	20	45.00
Line 4	3	1	8	8	20	68.33
Line 7	0	5	6	10	21	74.60
Line 8	1	4	14	6	25	66.67
WT	1	15	5	0	21	39.68
Line 4	0	0	11	10	21	82.54
Line 7	0	3	15	3	21	66.67
Line 8	0	9	7	5	21	60.31

Table S2. Primers names and their sequences used for vector construction in this study.

Olig name	Primer Abbreviation	Primer Sequence (5'-3')
CaSBP12	CaSBP12-VIGS-F	CGGGATCCATCCTCCGTTATGCTTTCTGGC
	CaSBP12-VIGS-R	GGGGTACCTACCTTGGGAATGGGTGAAACA
CaSBP12	CaSBP12-PBI121-F	GCTCTAGAATGTTGGACTATGACTGGGGAG
	CaSBP12-PBI121-R	CGGGATCCTGGTCTTTGCCTAAAACAATCC

Table S3. Primers names and their sequences used in this study for quantitative real-time PCR.

Olig name	Primer Abbreviation	Primer Sequence (5'-3')	Gene amplification length(bp)	Location of each primer
CaSBP12	RTCaSBP12-VIGS-F	GTTTCACCCATTCCCAAGGTAATT	213	exon
	RTCaSBP12-VIGS-R	TAGTACGTCGGTAAAGTCGATTAACAA		
CaActin2	CaActin2-F	TCCACCTCTTCACTCTCTGCTC	213	exon
	CaActin2-R	TGACCCATCCCTACCATAACAC		
CaAPX	CaAPX-F	AGAGGACAAGCCAGAACCAC	271	exon
	CaAPX-R	CCTTGTCTGATGGCAACTGT		
CaCAT2	CaCAT2-F	GAAGCCAAATCCTAAGTCCC	258	exon
	CaCAT2-R	CCAACTCGGATTGCCTCTT		
CaSOD	CaSOD-F	TATGGAGCCTTAGAACCTGC	173	exon
	CaSOD-R	CCATTGAACTTGATAGCACCT		
CaPOD	CaPOD-F	TCCTCCTCCTACTTCTAACC	302	exon
	CaPOD-F	ACAGACCTCTTTTGCTCACT		
CaSOS1	CaSOS1-F	GTTCGTGTCTCGTTTCCGC	163	exon
	CaSOS1-R	TCAAATCGGTCTGAACAGCATC		
CaHKT2-1	CaHKT2-1-F	GGCATTCATATCAGTTCAGTTTGT	231	exon
	CaHKT2-1-R	TTATCAACAGGCAAAAAAGTAGTAGAG		
CaHKT2-2	CaHKT2-2-F	AAACACAGTGTATTGCAGAACAACGAT	77	exon
	CaHKT2-2-R	TCAAGATTATGAAGACCTTCACCATTA		
Nbactin-97	Nbactin-F	TATGGAAACATTGTGCTCAGTGG	218	Exon
	Nbactin-R	CCAGATTCGTCATACTCTGCC		
NbAPX	NbAPX-F	CCAAGGGTTCTGACCATCTG	304	exon
	NbAPX-R	GCATAGTCGGCAAAGAAAGC		
NbCAT1	NbCAT1-RT-F	TCTATTGTGGTTCCAGGGGTTT	375	exon
	NbCAT-RT-R	CACCCACCGACGAATAAAGC		
NbSOD	NbSOD-RT-F	GCAGACGGACCTTAGCAACA	230	exon
	NbSOD-RT-R	TGGCGACGGTAGGAGCAT		
NbPOD	NbPOD-RT-F	AGGCTCAGGGGACAACAACT	194	exon
	NbPOD-RT-R	TCACAAAATCAGTGGCGAAA		
NbSOS1	NbSOS1-F	TCCCTTGGGGCAGTGG	200	exon
	NbSOS1-F	GCTACAGCTGAGTAGAACATCCC		
